# Exploring the relationship between retinal damage and cognition in multiple sclerosis: a cross-sectional OCT-MRI study

**DOI:** 10.1007/s10072-026-09157-3

**Published:** 2026-06-17

**Authors:** Riccardo Maria Borgo, Manuela Altieri, Alessandro Pasquale De Rosa, Rocco Capuano, Alvino Bisecco, Fabrizio Esposito, Alessandro Tessitore, Alessandro d’Ambrosio, Antonio Gallo

**Affiliations:** Department of Advanced Medical and Surgical Sciences, University of Campania “Luigi Vanvitelli”, Naples, Italy

**Keywords:** Multiple Sclerosis (MS), Cognitive impairment, Optical Coherence Tomography (OCT), Brain atrophy, Ganglion Cell Layer (GCL)

## Abstract

**Background:**

Cognitive impairment (CI) is a common and disabling feature of multiple sclerosis (MS), closely linked to brain neurodegeneration (e.g., brain atrophy). Optical coherence tomography (OCT) may offer a rapid and cost-effective method to evaluate neurodegeneration and its relationship with CI in MS.

**Objective:**

To explore the relationship between cognitive performance, retinal layer thickness, and brain volumetric measures in people with MS (pwMS), focusing on the relative contribution of individual retinal layers.

**Methods:**

In this cross-sectional study, 100 pwMS underwent: neurological and neuropsychological evaluation; brain 3T-MRI scan to compute brain volumes; spectral-domain OCT to assess peripapillary retinal nerve fiber layer (pRNFL), macular ganglion cell layer (GCL), inner plexiform layer (IPL), and combined ganglion cell-inner plexiform layer (GCIPL) thickness. Correlation, t-tests, and regression analyses were performed to explore the abovementioned associations.

**Results:**

Thinner retinal layers were associated with reduced brain volumes and lower cognitive performance. Among OCT-derived measures, GCL thickness showed the strongest association with both global cognitive Z-scores and domain-specific Z-scores for processing speed and executive functions. GCL also emerged as the only retinal predictor in regression models of cognitive outcomes. Classifying pwMS based on cognitive performance, cognitive impaired pwMS showed significantly lower GCL and pRNFL thickness compared to cognitive preserved pwMS.

**Conclusion:**

OCT-derived metrics of retinal damage linked to brain atrophy and CI in pwMS. Among retinal layers, GCL thickness is associated with both domain-specific and global cognitive performance and may potentially outperform GCIPL as an OCT-based marker of neurodegeneration, although findings should be interpreted with caution.

## Introduction

Multiple sclerosis (MS) clinical manifestations include not only a large variety of motor and sensory symptoms, but also cognitive impairment (CI), affecting 40–70% of patients [1]. The last decade has seen a heightened emphasis on MS-associated neurodegeneration, with researchers focusing on both the underlying pathological mechanisms and potential therapeutic strategies. The accumulation of progressive and permanent clinical disability (e.g., motor dysfunction and progressive ambulation restriction) is an established reflection of this process [2]. Methods capable of measure MS-associated neurodegeneration (i.e., permanent myelin and neuroaxonal loss) have been consistently explored to find reliable in-vivo procedures to monitor and predict disability accrual. In this context, MRI-derived measures of brain atrophy have been largely investigated and are now considered the gold standard, even if the translation to routine clinical practice (at a single subject level) still encounters some concerns and limitations [3]. Moreover, MRI scans are expensive and time-consuming, therefore the search of alternative methods has grown over the years. In this view, optical coherence tomography (OCT), a low-cost, non-invasive, and well-tolerated tool that finely measures retinal layers thickness (RLT), is under evaluation as a potential alternative to MRI. Indeed, since pivotal studies exploring the role of OCT in MS [4–8], besides the established use for the evaluation of optic neuritis (ON) [9], scientific literature has been investigating the OCT potential as a suitable predictor of relevant clinical and neuroradiological outcomes in MS. More specifically, OCT abnormalities have been shown to reflect different neurodegenerative substrates related to MS: (i) retrograde axonal and retinal ganglion cells loss following acute ON, (ii) primary retinal degeneration, and/or (iii) retrograde trans-synaptic degeneration following lesions in the optic pathways (from optic tract to visual cortex) [10]. Compared to healthy controls, peripapillary retinal nerve fiber layer (pRNFL) and macular ganglion cell/inner plexiform layer (GCIPL) have been found to be thinner in people with MS (pwMS) independently from previous ON clinical history. The inner nuclear layer (INL), inversely, has resulted thicker in the eyes of pwMS with previous ON than those without ON. Therefore, it has been postulated that pRNFL and GCIPL atrophy might be a proxy of neurodegeneration in MS, whilst INL thickening could be related with inflammatory disease activity [11]. Previous studies have investigated the relationship between OCT-derived parameters and structural MRI measures, such as white matter (WM) lesion volume and global and regional brain atrophy [4, 5, 8, 12–14], as well as cognitive performance [7, 15–19]. Indeed, since the pivotal study by Toledo et al. [7], which highlighted a correlation between pRNFL and cognitive tests evaluating verbal and visual memory, and executive and attention functions, further data have been published to investigate these findings, strengthened by OCT technical improvements (i.e., spectral domain OCT), as well as different approaches to neuropsychological testing, data collection, and statistical analysis. Notably, while Coric et al. [15] and Dreyer-Alster et al. [18] demonstrated strong associations between pRNFL and GCIPL atrophy and CI in large MS cohorts, other studies such as those by Frau et al. [16] and Baetge et al. [17] reported more selective or weaker associations. More recently, Cagol et al. [14] conducted a comprehensive study combining OCT-derived retinal measures, MRI-based brain volumetrics, and clinical outcomes, including physical disability and CI in pwMS, finding an association of pRNFL and GCIPL with clinical disability, performance at Symbol Digit Modality Test (SDMT), and brain structural damage, mainly reflected by gray matter (GM) atrophy and focal lesion burden, especially in the optic radiations. However, most of these studies have relied on composite retinal measures like GCIPL, which combine neuronal (ganglion cell) and synaptic (inner plexiform) layers, without dissecting the specific contribution of each layer to brain damage and cognitive outcomes. The separate analysis of the macular GCL, a purely neuronal structure, has been underexplored and may offer more precise insights into neuroaxonal degeneration associated with cognitive decline in MS.

Therefore, starting from this background, we designed this cross-sectional study aiming to further explore and clarify the relationship of retinal layer thickness, as measured by OCT, with brain volumetric measures and cognitive performance in pwMS. Specifically, we aimed to compare the relative contribution of individual retinal layers to cognitive outcomes, with a focus on distinguishing the predictive value of GCL thickness from that of the more commonly used GCIPL.

## Materials and methods

### Study design and population

In this cross-sectional study, a total of 120 pwMS were initially screened for eligibility at our MS Center, between June 2020 and December 2021. Inclusion criteria were: (i) a confirmed diagnosis of MS according to the 2017 McDonald criteria; (ii) absence of psychiatric comorbidities or major neurocognitive disorders as defined by the DSM-5; (iii) no clinical relapses and no use of corticosteroids or other medications known to affect cognitive function within 30 days prior to the evaluation. Exclusion criteria included: presence of ophthalmological comorbidities and/or refractive errors exceeding 6 diopters, due to their potential interference with OCT interpretation. Based on these criteria, 12 participants were excluded for concomitant ophthalmological conditions (e.g., glaucoma, pathological myopia), 5 for suboptimal OCT image quality, and 3 for recent corticosteroid use.

The remaining 100 pwMS completed the study protocol, which included: (i) neurological and neuropsychological evaluations, (ii) brain MRI, and (iii) spectral-domain OCT examination. A detailed medical and neurological history was obtained by two MS-trained neurologists. All participants were informed about the study procedures and objectives and provided written consent to participate. The study was approved by the Ethical Local Committee.

### Cognitive evaluation

To evaluate the cognitive performance of enrolled pwMS, the Italian version of Rao’s Brief Repeatable Battery of Neuropsychological Tests (BRB-N) was employed [20]. The BRB-N is a neuropsychological battery specifically validated for MS and it includes seven cognitive tests, administered in a fixed order, to assess verbal learning and memory (Selective Reminding Test; SRT), visuospatial learning and memory (10/36 Spatial Recall Test; SPART), sustained attention and information processing speed (SDMT, and Paced Auditory Serial Addition Test 3- and 2-s interval versions; PASAT-3″, PASAT-2″), and semantic verbal fluency (Word List Generation; WLG). In addition, the STROOP Color Word Interference Test was employed to evaluate inhibitory control. Using available normative data [20], Z-scores were calculated for each test and an overall cognition score was generated by computing the mean of all Z-scores.

### MRI assessment

Brain MRI scans were acquired on a 3.0 Tesla scanner equipped with a 32-channel parallel head coil (Discovery™ MR750, GE Healthcare, Milwaukee, Wisconsin). As part of the protocol, 3-dimensional (3D) T1 (gradient-echo sequence, orientation = sagittal, voxel size = 1 × 1 × 1, repetition time [TR] = 6.9 ms, echo time [TE] = 3 ms, inversion time [TI] = 650 ms, flip angle = 9, matrix size = 256 × 256) and Dual-Echo (DE; spin-echo sequence, orientation = axial, voxel size = 0.47 × 0.47 × 3, repetition time = 52.97 ms, echo time = 94 ms, flip angle = 111, matrix size = 384 × 512) sequences were acquired. All images were visually checked for quality assessment by an expert neuroradiologist. T2-hyperintense WM lesions were manually identified on T2 weighted images (extracted from DE scans) by two experienced neurologists, and T2-lesion volume (T2LV) was quantified using a semi-automatic local thresholding segmentation technique (Jim 8.0, Xinapse Systems Ltd, Colchester, UK). Brain volumetric measures, normalized for subject head size, were estimated with SIENAX [21] and FIRST [22], both part of FSL [23], on 3DT1 refilled images. The following measures were calculated: normalized brain volume (NBV), normalized gray matter volume (NGMV), normalized white matter volume (NWMV) and global thalamic volume (TV).

### OCT assessment

OCT examination was performed for both eyes of each participant by two trained neurologists using a Heidelberg-Spectralis spectral domain device (Heidelberg Eye Explorer, version 1.10.4.0) under dimmed light conditions, without pharmacological pupil dilation. pRNFL thickness was measured using a 3.7 mm circular ring scan centered on the optic nerve head (12°, 1536 A-scans, ART 100 frames). Thickness of GCL, IPL, and their composite value (GCIPL) were evaluated using a macular volume scan, with eye-tracking (20° x 20°, 25 vertical B-scans, 1024 A-scans per B-scan). Thickness values were obtained from the average of the sectors defined by the 1-, 3-, and 6-mm ETDRS grid centered on the fovea. For each patient, the mean value of both eyes was calculated for each retinal layer and used for all statistical analyses. The quality of every OCT scan was assessed according to OSCAR-IB Consensus Criteria for Retinal Quality Assessment [24]. Layers segmentation was performed automatically through the Heidelberg software implemented in the device; manual correction of segmentation errors was performed if necessary.

### Statistical analysis

Statistical analyses were performed by means of SPSS (IBM SPSS Statistics, version 25). A *p*-value ≤ 0.05 was considered the threshold of statistical significance. Pearson’s *r* coefficients were calculated to assess the possible association between cognitive performance (evaluated by Z-scores of each cognitive tests, and a cognitive global Z-score), pRNFL, GCL, IPL, and GCIPL mean thicknesses, and brain volumes. An independent samples t-test was performed to compare the thickness of pRNFL, GCL, IPL and GCIPL between cognitive preserved (CP group) and cognitive impaired (CI group) pwMS. Subjects showing at least two tests with a Z-score ≤ -1.5 SD in at least two different cognitive domains were allocated into the CI pwMS group, while the others were assigned to the CP pwMS group. Then, Benjamini-Hochberg correction for multiple comparisons was executed to reduce the likelihood on making a Type I error. Finally, several multiple regression analyses were computed to verify if pRNFL, GCL, IPL and GCIPL could predict cognitive performance. For the purposes of the regression analysis, the following dependent variables were considered: cognitive global Z-score (mean of each Z-score of every cognitive test), verbal memory Z-score (mean of Z-scores of SRT-LTS, SRT-CLTR, and SRT-D), visuo-spatial memory Z-score (mean of Z-scores of SPART and SPART-D), processing speed/attention Z-score (mean of Z-scores of SDMT, PASAT-2’’ and PASAT-3’’), verbal fluency Z-score (Z-score of WLG) and inhibitory control Z-score (Z-score of STROOP).

## Results

### Demographics, clinical, neuropsychological, OCT and MRI variables

Demographic, clinical, neuropsychological, OCT and MRI characteristics of the sample are summarized in Table 1.

**Table 1:** Demographic, clinical, neuropsychological, OCT and MRI characteristics of the sample

	MS patients (*n* = 100)
Mean Age (years, SD)	34.6 (10.5)
Sex (M/W)	33/67
Mean disease duration (months, SD)	73.1 (89.7)
Median EDSS (range)	1.5 (0–6.5)
Phenotype	CIS = 2 RIS = 6 RR = 81 PP = 4 SP = 7
pRNFL (µm, SD)	93.8 (13.9)
GCL (µm, SD)	38.1 (4.8)
IPL (µm, SD)	33.0 (3.3)
GCIPL (µm, SD)	71.2 (8.1)
T2LV (mm^3^, SD)	5589 (7352)
NBV (mm^3^, SD)	1506182.74 (89452)
NGMV (mm^3^, SD)	816680.77 (64668)
NWMV (mm^3^, SD)	689501.97 (56698)
Thalamus volume (mm^3^, SD)	10279.48 (1310)
Cognitive status (CP/CI)	67/33
SRT-LTS	− 0.47 (1)
SRT-CLTR	− 0.61 (1)
SRT-D	− 0.48 (1.12)
SPART	− 0.3 (1.04)
SPART-D	− 0.2 (0.92)
SDMT	− 0.01 (1.22)
PASAT 3’’	− 0.8 (1.22)
PASAT 2’’	− 0.82 (0.88)
WLG	− 0.99 (0.91)
STROOP	− 0.68 (2.4)
Global cognitive score	− 0.5 (0.78)

*Abbreviations*: *SD* Standard deviation, *EDSS* Expanded disability status scores, *pRNFL* Peripapillary retinal nerve fiber layer, *GCL* Ganglion cell layer, *IPL* Inner plexiform layer, *GCIPL* Ganglion cell–inner plexiform layer, *T2LV* T2 lesion volume, *NBV* Normalized brain volume, *NGMV* Normalized gray matter volume, *NWMV* Normalized white matter volume, *CP* Cognitive preserved, *CI* Cognitive impaired, *SRT-LTS* Selective reminding test – long term storage, *SRT-CLTR* Selective reminding test – consistent long term storage, *SRT-D* Selective reminding test – delayed recall, *SPART* Spatial recall test, *SPART-D* Spatial recall test – delayed recall, *SDMT *Symbol digit modalities test, *PASAT* Paced auditory serial addition test, *WLG* Word list generation, *STROOP* Stroop test

### Relationship between RLT and brain volumes

**Fig. 1:**
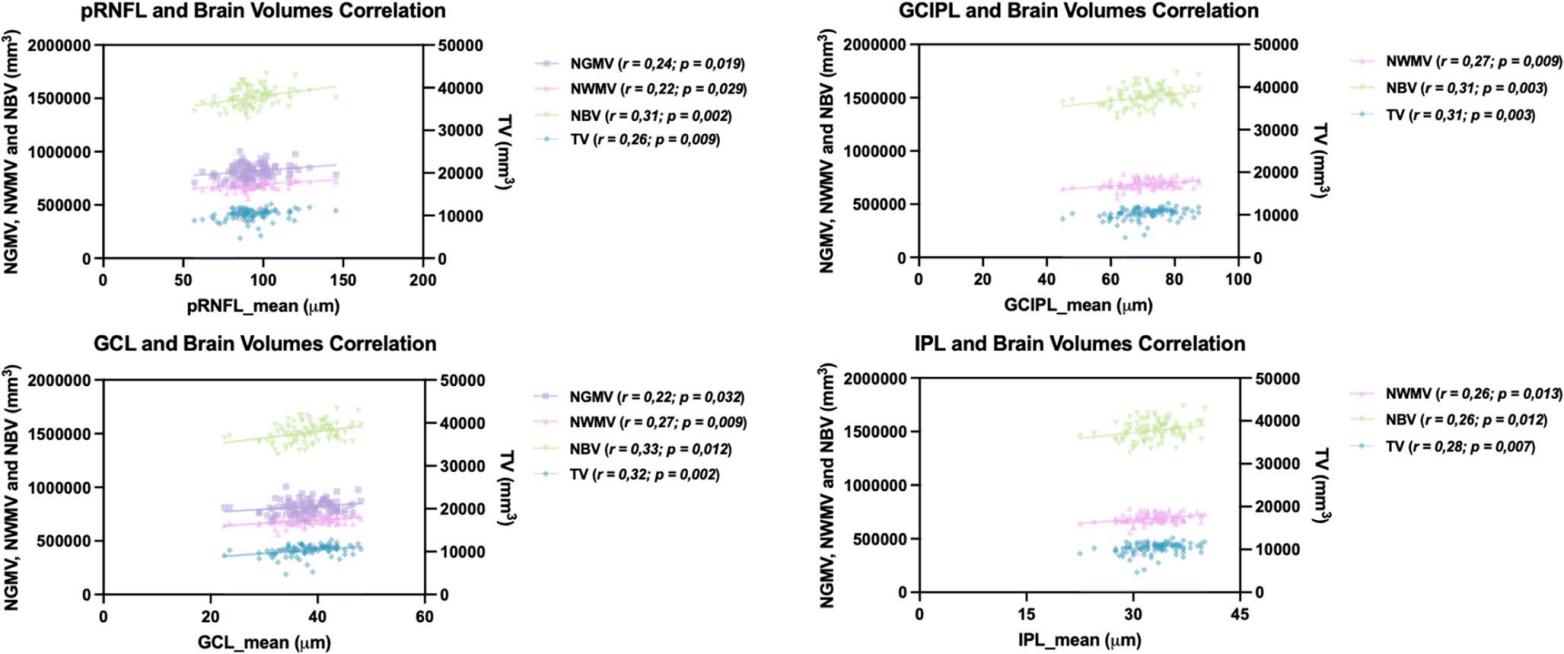
Correlation between OCT-derived retinal layer thickness and brain volumetric measures in people with MS. Scatter plots showing the association between mean thickness of pRNFL, GCIPL, GCL, and IPL with NGMV, NWMV, NBV, and TV. Pearson correlation coefficients (r) and p-values are reported for each pairwise comparison. Abbreviations: *OCT* Optical Coherence Tomography; *pRNFL* Peripapillary Retinal Nerve Fiber Layer; *GCIPL* Ganglion Cell–Inner Plexiform Layer; *GCL* Ganglion Cell Layer; *IPL* Inner Plexiform Layer; *NGMV* Normalized Gray Matter Volume; *NWMV* Normalized White Matter Volume; *NBV* Normalized Brain Volume; *TV* Thalamus Volume

RLT were consistently correlated with T2LV and brain atrophy measures considered in our study (NBV, NGMV, NWMV, and TV). Specifically, we found significant correlations between pRNFL with T2LV (*r* = − 0.31; *p* = 0.002), NBV (*r* = 0.31; *p* = 0.002), NGMV (*r* = 0.24; *p* = 0.019), NWMV (*r* = 0.22; *p* = 0.02), and TV (*r* = 0.26; *p* = 0.009), between GCIPL with T2LV (*r* = − 0.31; *p* = 0.002), NBV (*r* = 0.31; *p* = 0.003), NWMV (*r* = 0.27; *p* = 0.009), and TV (*r* = 0.31; *p* = 0.003), but also between GCL with T2LV (*r* = − 0.34; *p* = 0.001), NBV (*r* = 0.33; *p* = 0.01), NGMV (*r* = 0.22; *p* = 0.03), NWMV (*r* = 0.27; *p* = 0.009), and TV (*r* = 0.32; *p* = 0.002), and between IPL with T2LV (*r* = − 0.27; *p* = 0.009), NBV (*r* = 0.26; *p* = 0.01), NWMV (*r* = 0.26; *p* = 0.01), and TV (*r* = 0.28; *p* = 0.007) (Fig. 1).

### Relationship between T2LV and brain volumes with cognitive measures

As expected, a close relationship between brain volumes and cognitive performance was found. Specifically, all considered MRI measures (T2LV, NBV, NGMV, NWMV, TV) were consistently correlated with SPART and SDMT scores. In addition, T2LV was also significantly correlated to SPART-D, PASAT-3”, PASAT-2”, WLG, and STROOP scores, whereas NBV was correlated with SRT-CLTR, SRT-D, SPART-D, and STROOP. A significant correlation was also found between NGMV and SPART-D, whereas NWMW was significantly associated with STROOP. Finally, positive and significant correlations were found between TV with SPART-D and STROOP scores. The correlation analysis of T2LV and brain volumes, except for NGMV, showed a significant correlation with the cognitive global Z-score (Table 2). By employing the classification method to discriminate CI vs. CP pwMS (at least two tests with a Z-score ≤ − 1.5 SD), T2LV was significantly higher while brain volumes were significantly lower in CI pwMS compared to CP pwMS, and these differences were confirmed after correction with Benjamini-Hochberg method for multiple comparisons (Table 3).

**Table 2:** Correlation between MRI volumetric measures and cognitive test performance in people with MS

Cognitive Test	T2LV	NBV	NGMV	NWMV	TV
SRT-LTS	− 0.16	0.19	0.16	0.12	0.15
SRT-CLTR	− 0.13	0.21*	0.19	0.10	0.13
SRT-D	− 0.19	0.21*	0.14	0.17	0.20
SPART	− 0.32**	0.35**	0.27**	0.24*	0.24*
SPART-D	− 0.39**	0.34*	0.30**	0.20	0.21*
SDMT	− 0.43**	0.42**	0.33**	0.29**	0.34**
PASAT-3”	− 0.25*	0.07	− 0.02	0.13	0.03
PASAT-2”	− 0.27**	0.02	− 0.10	0.13	− 0.02
WLG	-0.21*	0.12	0.05	0.13	0.12
STROOP	− 0.42**	0.36**	0.20	0.31**	0.37**
Cognitive global Z-score	− 0.39**	0.32**	0.18	0.29**	0.25*

Pearson correlation coefficients (*r*) showing the associations between brain MRI volumetric measures and individual cognitive test Z-scores as well as cognitive global Z-score in people with pwMS. Statistical significance is indicated as follows: * = *p* < 0.05; ** = *p* < 0.01. Abbreviations: *T2LV* T2 Lesion Volume; *NBV* Normalized Brain Volume; *NGMV* Normalized Gray Matter Volume; *NWMV* Normalized White Matter Volume; *TV* Thalamus Volume; *SRT-LTS* Selective Reminding Test – Long Term Storage; *SRT-CLTR* Selective Reminding Test – Consistent Long Term Storage; *SRT-D* Selective Reminding Test – Delayed Recall; *SPART* Spatial Recall Test; *SPART-D* Spatial Recall Test – Delayed Recall; *SDMT* Symbol Digit Modalities Test; *PASAT* Paced Auditory Serial Addition Test; *WLG* Word List Generation; *STROOP* Stroop Test

**Table 3:** Comparison of OCT and MRI Measures between Cognitive Preserved (CP) and Cognitive Impaired (CI) pwMS Groups

Measures	CP group mean (SD) *N* = 67	CI group mean (SD) *N* = 33	t-statistic	*p*-value	*p*-value_adj_
OCT measures					
pRNFL (µm, SD)	95.9 (11.9)	89.5 (16.7)	2.20	**0.030**	**0.045**
GCL (µm, SD)	38.8 (4.2)	36.6 (5.8)	2.14	**0.035**	**0.045**
IPL (µm, SD)	33.4 (3.0)	32.2 (3.9)	1.60	0.114	0.114
GCIPL (µm, SD)	72.2 (7.1)	68.9 (9.7)	1.94	0.056	0.063
MRI measures					
T2LV (mm^3^, SD)	3613.4 (4204.0)	9795.5 (10384.4)	− 4.20	**< 0.001**	**< 0.001**
NBV (mm^3^, SD)	1529267.3 (85596.2)	1457780.0 (78311.3)	3.93	**< 0.001**	**< 0.001**
NGMV (mm^3^, SD)	828097.0 (59971.0)	792743.5 (68527.2)	2.56	**0.011**	**0.012**
NWMV (mm^3^, SD)	701170.2 (49294.0)	665036.2 (63894.1)	3.04	**0.003**	**0.007**
TV (mm^3^, SD)	10547.0 (1061.2)	9718.6 (1596.7)	3.02	**0.003**	**0.007**

Group-level differences assessed using independent-sample t-tests. Statistically significant differences (*p* < 0.05) are reported in bold. Values are expressed as mean ± standard deviation unless otherwise indicated

*Abbreviations*: *CP* Cognitive preserved, *CI* Cognitive impaired, *SD* Standard deviation, *OCT* Optical coherence tomography, *MRI* Magnetic resonance imaging, *pwMS* People with multiple sclerosis, *pRNFL* Peripapillary retinal nerve fiber layer, *GCL* Ganglion cell layer, *IPL* Inner plexiform layer, *GCIPL* Ganglion cell–inner plexiform layer, *T2LV* T2 lesion volume, *NBV* Normalized brain volume, *NGMV* Normalized gray matter volume, *NWMV* Normalized white matter volume, *TV* Thalamus volume

### Relationship between RLT and cognitive measures

**Fig. 2:**
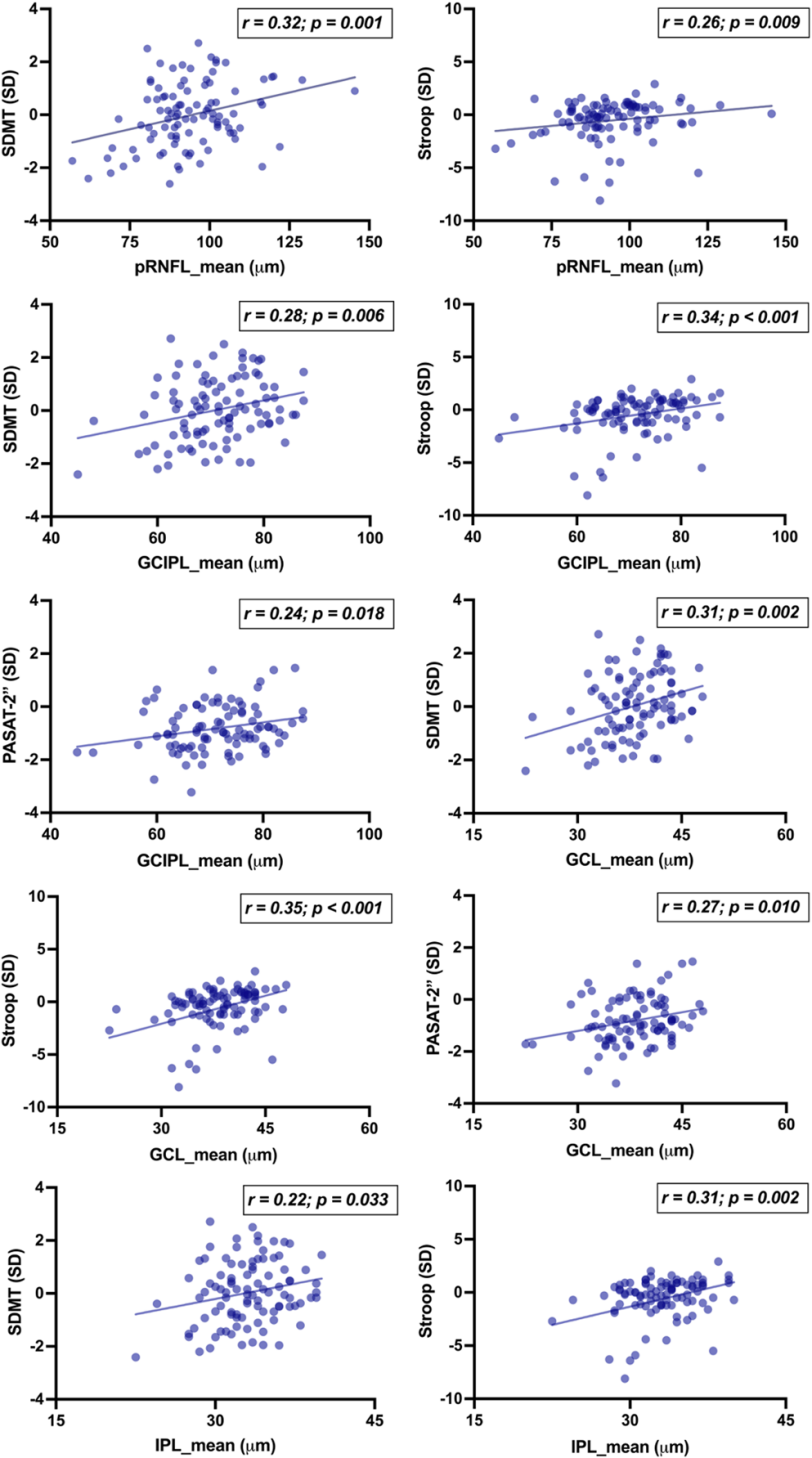
Correlation between OCT-derived retinal layer thickness and domain-specific cognitive scores. Scatter plots illustrating the significant relationship between OCT-derived retinal layer thickness (pRNFL, GCIPL, GCL, IPL) and cognitive test scores in pwMS, including SDMT, STROOP test, and PASAT-2″. Pearson’s r and p-values are shown. Abbreviations: *OCT* Optical Coherence Tomography; *pRNFL* Peripapillary Retinal Nerve Fiber Layer; *GCIPL* Ganglion Cell–Inner Plexiform Layer; *GCL* Ganglion Cell Layer; *IPL* Inner Plexiform Layer; *SDMT* Symbol Digit Modalities Test; *STROOP* Stroop Test; *PASAT* Paced Auditory Serial Addition Test

**Fig. 3:**
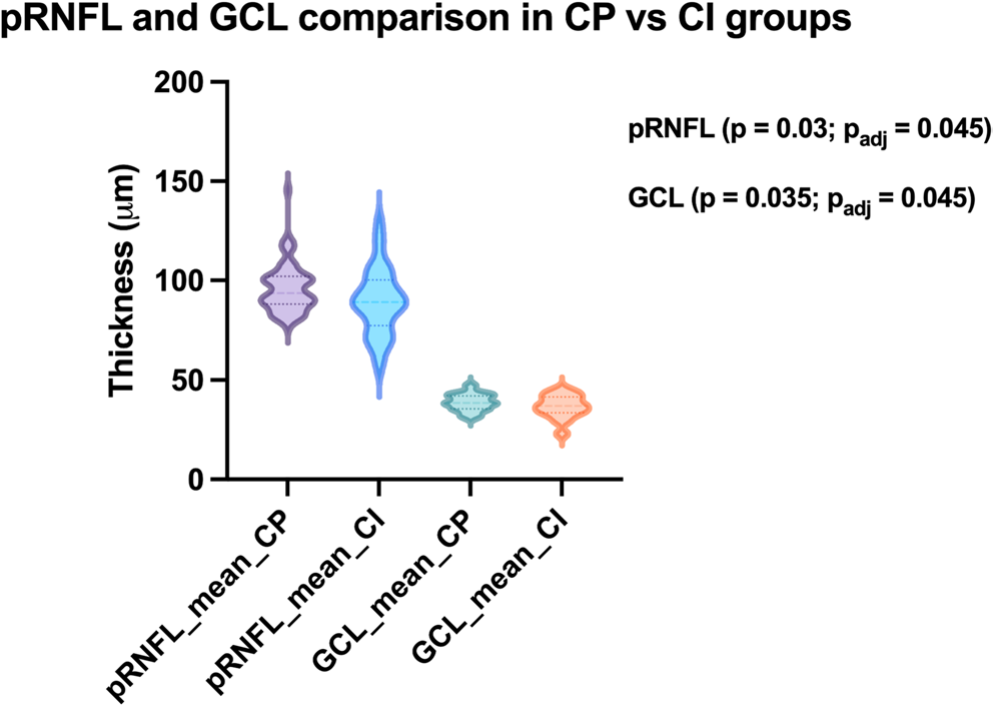
Comparison of pRNFL and GCL thickness between cognitive preserved and cognitive impaired pwMS. Violin plots showing the distribution of pRNFL and GCL mean thickness values in cognitive preserved versus cognitive impaired pwMS. Statistically significant differences were observed for both pRNFL (*p* = 0.03; p_adj = 0.045) and GCL (*p* = 0.035; p_adj = 0.045) thickness between groups. Abbreviations: *pRNFL* Peripapillary Retinal Nerve Fiber Layer; *GCL* Ganglion Cell Layer; *CP* Cognitive Preserved; *CI* Cognitive Impaired; *pwMS* people with Multiple Sclerosis

The correlation analysis revealed significant positive results between all RLT and SDMT (pRNFL: r = 0.32, p = 0.001; GCIPL: r = 0.28, p = 0.006; GCL: r = 0.31, p = 0.002; IPL: r = 0.22, p = 0.033) and STROOP (pRNFL: r = 0.26, p = 0.009; GCIPL: r = 0.34, p = 0.001; GCL: r = 0.35, p < 0.001; IPL: r = 0.31, p = 0.002). Moreover, GCIPL, GCL and IPL showed an additional significant correlation with PASAT-2” (*r* = 0.24, *p* = 0.018; *r* = 0.27, *p* = 0.010; *r* = 0.21, *p* = 0.047, respectively). These results are reported in Fig. 2. A significant correlation was also found between GCIPL and GCL thickness with the cognitive global Z-score (*r* = 0.27, *p* = 0.010; *r* = 0.30, *p* = 0.004, respectively). After Benjamini-Hochberg correction, the comparison of RLT in CI vs. CP groups (classified according to the method previously described in this paper) revealed higher pRNFL (*p* = 0.030; p_adj_ = 0.045) and GCL (*p* = 0.035; p_adj_ = 0.045) thickness in the CP group (Fig. 3; Table 3). The regression analysis revealed that, among the RLT considered as independent predictors of the various composite cognitive Z-scores, GCL thickness was the only significant predictor for cognitive global (β = 0.30, *p* = 0.004), inhibitory control (β = 0.35, *p* < 0.001), and processing speed/attention (β = 0.29, *p* = 0.004) Z-scores. No additional prediction models emerged when considering the other RLT, and none of the possible RLT combination models were able to predict verbal and visuo-spatial memory Z-scores, and verbal fluency Z-score

## Discussion

Our results showed a relationship between retinal damage, as measured by pRNFL and GCIPL thinning - with more consistent results achieved by GCL alone - and worse cognitive performance on neuropsychological tests assessing information processing speed and executive functions. The analysis of the relationship between OCT measures and brain volumes revealed a significant association of the RLT with the brain volumetric measures considered in the present study. Finally, brain volumes showed an association with cognitive performance in many cognitive domains, with results consistent with previous published data [25].

Despite the evidence of mixed results arising from the available literature on the association between OCT measures and cognitive performances, as described in a recent systematic review and meta-analysis [19], our findings are in line with most of the previous studies showing a link between retinal damage and impaired cognitive performance measured by neuropsychological batteries specifically validated for MS population. This association was confirmed, in particular, for the SDMT (assessing information-processing speed performance), which represents the most frequently administered test both in research studies and clinical practice, as it has proven to be a quick, easy, and sensitive measure in the evaluation of cognitive performance in pwMS [26], as well as a valuable/efficient/accurate predictor of the global cognitive status measured with comprehensive, MS-validated, neuropsychological batteries [27].

A key finding of our study is that the GCL, when analyzed independently, showed stronger associations compared to the more commonly used composite GCIPL thickness in predicting both global cognitive performance and specific domains such as processing speed and executive function. This suggests that GCL, as a purely neuronal structure, may provide more direct insight into neuroaxonal degeneration, while the inclusion of synaptic components (i.e., inner plexiform layer) in GCIPL may introduce variability not directly related to cognitive decline. While most previous studies employed GCIPL as a surrogate marker, our direct comparison may highlight the potential of GCL thickness to serve as a more specific and sensitive OCT-based biomarker of cognitive dysfunction. However, these findings should be interpreted considering the heterogeneity of the study population, including the presence of progressive MS phenotypes.

Our study expanded available evidence supporting the relationship between RLT, cognitive performance, and brain structural integrity. Previous findings showed the potential different, but complementary, role of WM and GM damage underlying CI in MS: while WM damage and volumes were found to be the best predictors of processing speed and working memory, GM damage and volumes scored better in predicting verbal memory, euphoria, and disinhibition [28]. So, alongside WM involvement, cortical and sub-cortical GM damage is considered just as much critical in CI pathogenesis in pwMS, highlighting the complexity of the pathological processes underlying CI in MS and the difficulty in their in-vivo detection. In this sense, based on our results, OCT alterations might reflect more WM and thalamic rather than cortical/global GM damage in cognitively impaired pwMS [29], corroborating the results of some of the previous studies [30, 31]. Interestingly, indeed, OCT variables did not only correlate with visual (i.e., SDMT and STROOP), but also to non-visual tests (i.e., PASAT) and global cognitive performance. These results may suggest that our findings are independent from (very common) visual pathways damage and further corroborates previous studies that found an association between OCT measures and cognitive performance on tests that not rely just on visual components [19]. Finally, although there’s still a need to better define appropriate cut-off values for single subject analysis, our results have shed light on a potential future implication for MS routine evaluation, as they provide further evidence to encourage clinicians to consider implementing OCT in their MS clinical practice since it is a less expensive (respect to MRI), well-tolerated and rapid diagnostic instrument able to generate measures correlated with both structural brain damage (detectable using MRI) and cognitive impairment (detectable trough a time-consuming cognitive evaluation).

Some limitations must be acknowledged. First, we did not exclude eyes with a history of ON, as this condition represents a frequent and clinically relevant component of retinal pathology in MS. Including such eyes allowed us to preserve sample representativeness and avoid selection bias; however, the lack of a sub-analysis taking into account previous ON, as done in other studies [12], could be interpreted as a limitation of this study. Additionally, a small proportion of patients in our cohort (approximately 10%) presented progressive MS phenotypes (primary and secondary progressive MS). Given that these forms are typically characterized by a higher degree of neurodegeneration, retinal thinning and worse cognitive performance, their inclusion may have influenced the observed associations between retinal measures and cognitive performance. However, we chose not to exclude these patients to preserve the representativeness of a real-world MS population and avoid selection bias. Nevertheless, this aspect should be considered when interpreting our findings; therefore, future studies may evaluate the association between retinal damage and cognition, with larger sample sizes and a focus on potential differences across MS phenotypes. Second, although we did not include a healthy control group, our aim was to focus on within-cohort variability in OCT and MRI measures as they relate to cognitive status in a real-world MS population. Third, the cross-sectional design precludes any causal interpretation of our findings and limits conclusions regarding the longitudinal predictive power of OCT metrics. Despite these limitations, our study has several strengths, including a well-characterized clinical cohort, the use of validated neuropsychological assessments, high-resolution MRI and OCT protocols, and a direct comparison of individual retinal layers. Taken together, our findings suggest that OCT-derived retinal measures may be a reliable and clinically applicable biomarker for cognitive impairment in MS. Given its non-invasive, cost-effective, and rapid nature, OCT – particularly with focused analysis on the GCL – may support early detection of cognitive dysfunction and complement MRI in routine neurological practice.

Future research should also explore the integration of OCT measurements with other clinical and neuroimaging markers, including advanced MRI-derived metrics of cortical and subcortical GM integrity, functional connectivity, and brain network efficiency. Such multimodal approaches may offer a more comprehensive understanding of the complex mechanisms underlying CI in MS.

Moreover, the development of automated and standardized OCT pipelines, possibly integrated with machine learning algorithms, could enhance the sensitivity and specificity of retinal biomarkers for early cognitive screening and eventually become markers to support personalized cognitive profiling and risk stratification in routine MS care.

In summary, our study supports previous evidence suggesting that retinal damage, as assessed by OCT, is associated with both brain structural changes and cognitive impairment in people with MS. Among the retinal layers analyzed, GCL thinning appeared to be the most informative marker, not only for processing speed and executive function, but also for overall cognitive performance. These results may further underscore the potential of OCT, and specifically GCL analysis, as a rapid, low-cost, well-tolerated, and non-invasive tool for cognitive assessment in clinical MS practice, although they should be interpreted with appropriate caution.

## Data Availability

The data that support the findings of this study are not openly available due to reasons of sensitivity and are available from the corresponding author upon reasonable request. Data are located in controlled access data storage at University of Campania Luigi Vanvitelli.
